# Permanent visual impairment following a Behçet’s disease flare while on calcitonin gene-related peptide receptor antagonist therapy: a case report

**DOI:** 10.1186/s12886-025-04322-2

**Published:** 2025-09-30

**Authors:** Fawad A. Khan, Alaa Malik, Evan Nelson, Kian Fahimdanesh, Karmveer Kaur, Jasmine Elison, Mohamed Sayed

**Affiliations:** 1 The McCasland Family Comprehensive Headache Center, Department of Neurology, Ochsner Neuroscience Institute, Ochsner Health, 1514 Jefferson Highway, New Orleans, LA USA; 2 The University of Queensland Medical School, Brisbane, Australia; 3 Louisiana State University Health Sciences Center School of Medicine, New Orleans, LA USA; 4 Retina and Vitreous Specialists New Orleans, New Orleans, LA USA; 5 Department of Cell Biology & Anatomy, Louisiana State University Health Sciences Center, New Orleans, LA USA

**Keywords:** Behçet’s disease, Migraine, Vision loss, Vasodilation, Calcitonin Gene-Related peptide receptor, CGRP antagonist, Erenumab

## Abstract

**Background:**

Behçet’s disease (BD) is a chronic, relapsing, systemic vasculitis that can involve both arteries and veins. Ocular involvement, including non-granulomatous panuveitis and occlusive retinal vasculitis, is common and a significant cause of morbidity. Erenumab is a monoclonal antibody targeting the calcitonin gene-related peptide (CGRP) receptor approved for migraine prevention Although it is generally well tolerated, recent concerns have emerged regarding its vasoconstrictive potential in patients with underlying vascular disorders.

**Case presentation:**

We report a case of a 44-year-old woman with a history of BD, well-managed with azathioprine and methotrexate, who developed painless, bilateral subacute visual loss eleven days after receiving her second monthly dose of erenumab. This occurred during a BD flare marked by oral ulcers and severe migraine. Despite corticosteroid treatment, visual acuity did not improve. Erenumab was discontinued, but at her following visit four weeks later, vision remained impaired and visual fields showed blind spot enlargement and nonspecific defects.

**Conclusion:**

We hypothesize that the patient’s visual loss was due to secondary ischemia from BD–associated small-vessel vasculitis, potentially exacerbated by CGRP receptor blockade from erenumab, which may have impaired compensatory vasodilation in the optic nerve or retinal circulation. This case represents the first report of permanent bilateral visual impairment associated with CGRP antagonist use in a patient with BD. Recent FDA labeling updates for CGRP-targeting agents now caution against use in patients with preexisting vascular disease, including risk of hypertension and Raynaud’s phenomenon. This case underscores the need for heightened caution when prescribing CGRP inhibitors to patients with systemic vasculitic disorders and highlights the importance of further investigation to better define the safety profile of these agents in individuals with underlying vascular disease.

## Background

Behçet’s disease (BD) is a chronic, systemic inflammatory disorder characterized by widespread vasculitis, which affects both arteries and venules [1]. Ocular manifestations are common in BD, with non-granulomatous panuveitis and occlusive necrotizing retinal vasculitis being the most frequent, often leading to vision impairment [2]. Other rare complications include optic neuritis and perineuritis. Additionally, migraine headaches are frequently reported in individuals with BD [3]. 

Erenumab, a monoclonal antibody (mAb) targeting the calcitonin gene-related peptide (CGRP) receptor, is widely used for migraine prevention. According to a recent position statement by the American Headache Society, CGRP-targeting therapies, including erenumab, are considered first-line options due to their established efficacy, safety, and tolerability [4]. However, recent reports have highlighted potential ischemic risks in individuals with preexisting vascular conditions, as the inhibition of CGRP’s vasodilatory effects may impair blood flow and increase the risk of adverse vascular events in susceptible patients [5, 6]. 

Vision loss associated with erenumab has not been reported in clinical trials or long-term safety studies, and no cases of visual impairment have been documented in patients who experienced elevated blood pressure during treatment [5]. However, a single case of unilateral vision loss was reported, attributed to Susac’s syndrome. This occurred nine months after initiating erenumab. The vision loss was attributed to platelet aggregation and thrombus formation in a retinal artery branch, a process likely influenced by the inhibition of the CGRP pathway [7]. 

### Case presentation

Our patient is a 44-year-old female with a history of BD characterized by recurrent oral ulcerations treated with azathioprine and methotrexate, anxiety, asthma, cervical disc disease, and chronic migraines. Monthly subcutaneous injections of erenumab 70 mg were initiated for migraine prevention. Eleven days after her second injection, she experienced a flare-up of BD, presenting with oral ulcers, severe migraine, and subacute, painless bilateral blurry vision, along with monocular diplopia in her left eye.

An optometric evaluation revealed normal intraocular pressures, full confrontational visual fields, and no ophthalmoplegia. Visual acuity measured 20/60–1 in the right eye and 20/400 in the left eye, reflecting a significant subjective decline of approximately 50% from her usual baseline, although prior objective visual acuity data were unavailable for comparison. The external structures, lids and lashes, conjunctiva, sclera, cornea, anterior chamber, iris, pupil, lens, and anterior vitreous were all normal, with clear structures, no signs of redness, swelling, discharge, inflammation, or opacities, and pupils equal and reactive to light. An afferent pupillary defect was not assessed. Optical coherence tomography (OCT) demonstrated a normal, well-defined foveal contour with intact retinal layers, no intraretinal or subretinal fluid, no macular edema, a normal optic nerve head configuration without swelling or abnormal cupping, and retinal nerve fiber layer thickness within normal limits without focal thinning or defects. Fluorescein angiography of both eyes demonstrated normal retinal and choroidal perfusion with no leakage, staining, blockage, neovascularization, microaneurysms, or capillary nonperfusion; optic disc and macular circulation appeared normal bilaterally. Neurological exam was normal. Despite a short course of steroid eye drops for 1 week, no improvement in vision was reported by the patient.Brain magnetic resonance imaging and angiography as well as cerebral angiography ruled out secondary causes of vision loss, including central nervous system vasculitis, neuroinflammatory conditions, cerebral venous sinus thrombosis, and ischemic etiologies. Serum inflammatory markers were within normal limits. Empirical treatment with oral steroids (prednisone 60 mg daily) for 30 days was initiated, leading to resolution of oral ulcers and improvement of migraine. However, the patient’s vision remained subjectively unchanged. Erenumab was discontinued.

**Fig. 1:**
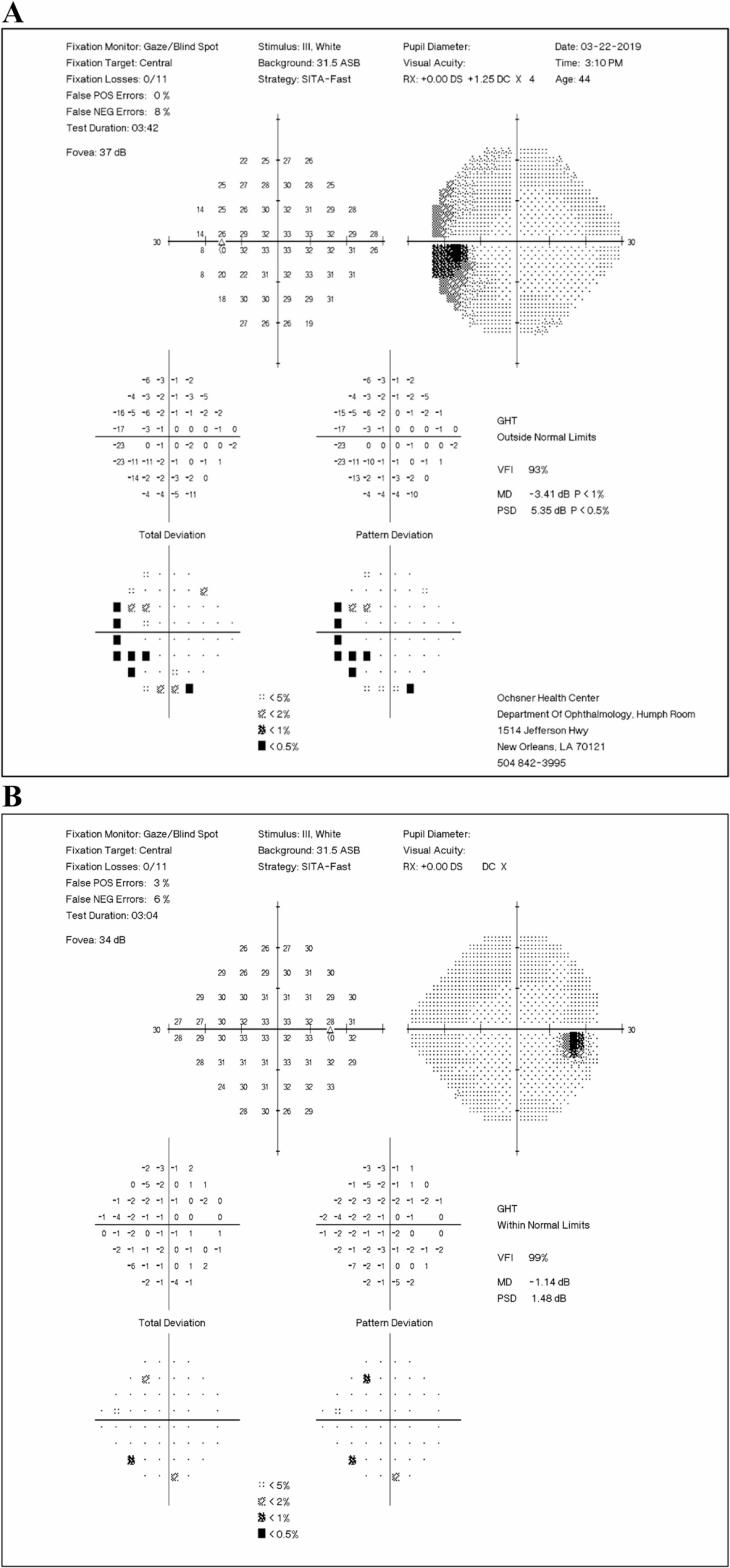
Humphrey 24-2 visual field testing showing enlarged blind spot of the left eye and mild nonspecific defects of the right eye (**A** – left eye. **B –** right eye)

A follow-up evaluation at 4 weeks revealed visual acuities of 20/80 − 1 in the right eye and 20/100 in the left eye, with no evidence of a relative afferent pupillary defect. Formal visual field testing showed enlargement of the left eye’s blind spot and mild nonspecific defects in the right eye. See Fig. 1A and B. OCT indicated normal and symmetric retinal nerve fiber layer and normal macular architecture in both eyes. Fluorescein angiography was unremarkable.

At 48 months, there was no improvement in visual acuity. At the most recent follow-up at 70 months, the visual impairment remained unchanged, though the patient reported no new BD–related symptoms. Lifestyle adaptations included maintaining full-time employment, using high-powered glasses, and working with extra-large computer monitors to accommodate the visual deficit.

## Discussion

Blockade of CGRP receptor–mediated vasodilation, particularly in tissues with a high density of these receptors, may increase the risk of ischemic events—raising concerns for patients with pre-existing conditions such as myocardial infarction, unstable angina, or poorly controlled hypertension [8]. In our patient, we hypothesize that the permanent visual impairment in both eyes was likely triggered during a flare of BD. The associated small-vessel vasculitis may have predisposed the optic nerves to reduced perfusion, which was further exacerbated by impaired vasodilatory response due to CGRP receptor blockade—ultimately contributing to secondary ischemia, possibly involving the retrobulbar segments of the optic nerves [1, 2]. 

Notably, imaging did not reveal any large-vessel involvement, supporting the likelihood of diffuse small-vessel pathology affecting both eyes. This pattern is distinct from typical BD-related optic neuritis, which usually presents with hyperemic or swollen optic discs, peripapillary hemorrhages, significant intraocular inflammation, or retinal vasculitis—none of which were present in our case. Additionally, structural changes of the optic nerve head and retinal vasculitis typical of BD were absent on multimodal imaging.

The visual impairment occurred shortly after the second dose of erenumab, and given the known pharmacokinetics of mAb injections—monthly administration with a long half-life—we hypothesize that the cumulative pharmacologic effect following the second dose may have crossed a physiological threshold, leading to compromised ocular perfusion [9]. Furthermore, no improvement in visual function was observed despite corticosteroid therapy.

Our case represents the first reported observation of permanent visual sequelae in a patient with BD receiving a calcitonin gene-related peptide antagonist therapy. While caution is warranted in generalizing these findings to all patients with underlying ocular vascular disease or ocular involvement in BD, this case raises an important safety concern. Notably, on March 21, 2025, the US Food and Drug Administration (FDA) issued a Drug Safety Labeling Change for the class of calcitonin gene-related peptide therapies—including both gepants and mAbs such as erenumab [10]. The updated labeling includes safety clauses on development of hypertension and worsening of pre-existing hypertension, and (b) development of Raynaud’s phenomenon and recurrence or worsening of pre-existing Raynaud’s phenomenon.

A balanced understanding of these therapies’ vascular risk is essential. Post-marketing surveillance and real-world data have signaled adverse events such as alopecia and Raynaud’s phenomenon for both anti-CGRP mAbs and gepants [11, 12]. Rare cerebrovascular events have also been described, including thalamic infarction after a single dose of erenumab (with confounders such as concurrent estrogen-containing oral contraceptives and triptan use) and reversible cerebral vasoconstriction syndrome in patients on fremanezumab or erenumab.

In contrast, large randomized controlled trials enrolling over 10,000 migraine patients have not demonstrated a significant increase in cardiovascular risk with either mAbs or gepants [13]. Concomitant use with vasoconstrictive agents, such as triptans or nonsteroidal anti-inflammatory drugs, has been well tolerated in both trial and real-world settings, supporting an overall favorable safety profile. However, what remains to be clarified is whether certain patient subgroups possess heightened susceptibility to ischemic complications despite this reassuring population-level safety.

Considering this update and our observations, we remain at a critical reconsideration point where the safety profile of CGRP antagonists—particularly their vascular effects and potential to cause ischemia in vulnerable organs—is still being elucidated. Clinicians should exercise caution when prescribing these therapies to patients with BD and other vasculitic conditions. Further research is needed to better understand the safety and risks of these agents in populations with underlying vascular diseases.

## Data Availability

No datasets were generated or analysed during the current study.

## Abbreviations

BD: Bechet’s disease
CGRP: Calcitonin gene-related peptide
mAb: Monoclonal antibodies
OCT: Optical coherence tomography
